# Hkakabo Razi landscape as one of the last exemplar of large contiguous forests

**DOI:** 10.1038/s41598-020-70917-y

**Published:** 2020-08-19

**Authors:** Marcela Suarez-Rubio, Grant Connette, Thein Aung, Myint Kyaw, Swen C. Renner

**Affiliations:** 1 Institute of Zoology, University of Natural Resources and Life Sciences, Gregor-Mendel-Strasse 33, 1180 Vienna, Austria; 2 Conservation Ecology Centre, Smithsonian Conservation Biology Institute, 1500 Remount Road, Front Royal, VA 22630 USA; 3 Myanmar Bird and Nature Society, 221/223 Shwegondine Road, Yangon, Myanmar; 4 Mount Popa National Park Headquarters, Popa, Myanmar; 5 Ornithology, Natural History Museum, Burgring 7, 1010 Vienna, Austria

**Keywords:** Ecology, Biodiversity, Conservation biology, Forest ecology, Tropical ecology, Environmental impact

## Abstract

Deforestation and forest degradation around the world endanger the functioning of ecosystems, climate stability, and conservation of biodiversity. We assessed the spatial and temporal dynamics of forest cover in Myanmar’s Hkakabo Razi Landscape (HRL) to determine its integrity based on forest change and fragmentation patterns from 1989 to 2016. Over 80% of the HRL was covered by natural areas, from which forest was the most prevalent (around 60%). Between 1989 and 2016, forest cover declined at an annual rate of 0.225%. Forest degradation occurred mainly around the larger plains of Putao and Naung Mung, areas with relatively high human activity. Although the rate of forest interior loss was approximately 2 to 3 times larger than the rate of total forest loss, forest interior was prevalent with little fragmentation. Physical and environmental variables were the main predictors of either remaining in the current land-cover class or transitioning to another class, although remaining in the current land cover was more likely than land conversion. The forests of the HRL have experienced low human impact and still constitute large tracts of contiguous forest interior. To ensure the protection of these large tracts of forest, sustainable forest policies and management should be implemented.

## Introduction

Despite human practices and the unprecedented use of natural resources, forests are still widely distributed globally and cover around 30% of the Earth’s surface^1,2^. However, ongoing deforestation and forest degradation jeopardize the functioning of biogeochemical and hydrological cycles, climate stability and conservation of biodiversity^3–5^. Net loss of forest area occurs largely in the tropics^6,7^ and this forest loss continues to impact areas with particularly high conservation value^8,9^.

Tropical forests play a key role in the global carbon cycle and support more than half of the world’s biodiversity^10^. Industrial logging, agricultural expansion, fire, mining/resource extraction and urban growth have led to extraordinary loss of tropical forest^11,12^. The amount of forest loss differs between continents^13^, with the highest levels occurring in South America and Asia^2^. In Southeast Asia, Myanmar had the second highest rate of net forest loss between 1990 and 2015, trailing only Indonesia, with a loss rate of 546,000 ha y^−1^ between 2010 and 2015^1^. Furthermore, this rate of forest loss represented a 25% increase since the 1990s. The driving forces behind the high rates of forest loss in Southeast Asia are logging and the global demand for crops such as oil palm, sugar, and wood fibre^14^.

Despite having the third largest annual forest loss in the world between 2010 and 2015^1^, Myanmar remains one of the most heavily forested countries in Southeast Asia^15,16^. Myanmar is the second largest exporter of Teak (*Tectona grandis*), a valuable timber species, and much of the rural population continues to depend on forests to supplement their livelihoods^17^. Some forest areas are used for small scale agroforestry and up to 77% of energy demands are covered by traditional energy sources such as fuel wood, charcoal and biomass^18^. Selective logging on government forest reserves has historically been managed under the Myanmar Forest Selection System, which sets harvest quotas to sustain long-term timber yields^19^. In unmanaged forests, though, logging concessions have far less oversight^20^ and contribute to the rapid loss of relatively intact forest. Besides wood extraction, agricultural expansion and infrastructure development are the most common causes of forest loss^21^. Nonetheless, Myanmar has retained much of its original forest cover, stretching across 63% of the country’s land^16^.

One of Myanmar’s largest remaining area of contiguous intact forest is found in the northern-most part of the country^16^. The Hkakabo Razi Landscape (HRL; also known as the Northern Forest Complex or Northern Mountain Forest Complex) has an exceptionally rich biodiversity and some of the highest concentrations of endemic species in the world^22,23^. New species are continuously being described, including plants^24–28^, mammals^29–31^ and birds^32–35^, described to be new for the HRL^36,37^ or reconfirmed to occur for the HRL^38^. In 2000, human settlements and paddyfields covered less than 14% of the area^39^, and changes in forest cover from 1990 to 2000 were minimal with an overall deforestation rate of < 1%^39^. Access to the area is limited, with most areas reachable only by walking trails as roads only exist close to towns in the southern part of the HRL^39^.

However, the remaining large forest tracts in Myanmar are under pressure^40^. The country’s recent political and economic reforms are attracting investors, leading to far-reaching changes in forestry and other land-based investments sectors^16,41–43^. Therefore, areas previously inaccessible are starting to open up for systematic, large-scale resource extraction, timber production, commercial plantations (MOECAF 2011) and extensive tourism^44^ as a way to achieve economic growth. Given these rapid developments, assessing the current forest distribution and quantifying the extent of forest loss and degradation in the HRL are required to understand the effects economic and development changes are having on this region, and to develop strategies for sustainable land management.

The objective of this study was to assess the spatial and temporal dynamics of forest cover in the HRL. We mapped land cover, including several ecologically-distinct forest types, using multi-temporal Landsat satellite imagery and quantified forest change and fragmentation patterns across a 27-year interval. We then identified major spatial predictors of forest change. Through this assessment, we aim to determine whether the forests of the HRL are still of high integrity^45^.

## Methods

### Study area

**Figure 1:**
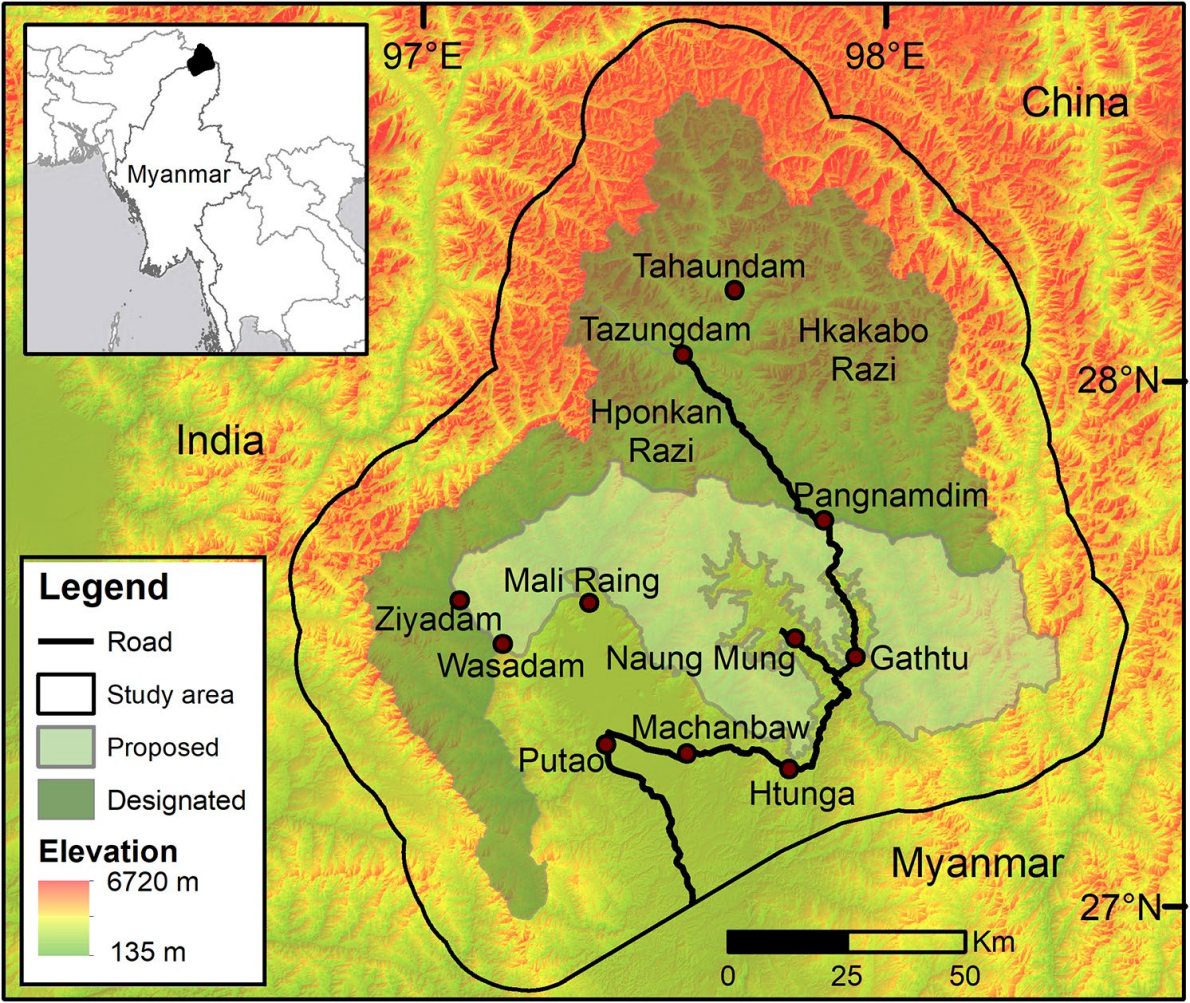
The Hkakabo Razi Landscape including protected areas, major settlements and elevation. The map was produced in ArcGIS v. 10.6.1 (https://desktop.arcgis.com).

Our study area was located in the northernmost tip of Kachin State, Myanmar (27°46′50.55″ N, 97°41′11.27″ E). It is surrounded by Yunnan/China to the east, Arunachal Pradesh/India to the west and the Tibetan Plateau to the north (Fig. 1). The HRL includes the Hkakabo Razi National Park (established in 1998), Hponkan Razi Wildlife Sanctuary (established in 2003), and the currently proposed, but not formally established, southern extension of the Hkakabo Razi National Park. The combined designated and proposed protected areas cover 11,820 km^2^. We added a buffer of 15 km around this landscape and included the Putao plains as part of our study area (Fig. 1).

The HRL is a transition zone between three major biodiversity hotspots^46^ and overlaps largely with three Endemic Bird Areas^47–49^—a unique arrangement worldwide making the area exceptional^46^. The highest peak is Mount Hkakabo Razi at 5,882 m a.s.l. and the lowest elevation is on the Putao plains at about 420 m. The entire landscape is known for its hyper-mesic conditions. Based on current high-resolution climate data from CHELSA^50^, annual rainfall ranges from around 4,000 mm at lower elevations to around 6,000 mm at higher elevations. The climate station in Putao measures a mean July temperature of 21 °C with an average temperature for the coldest month (January) of around 5 °C. The summits of Hponkan Razi and Hponyin Razi, both located in the Hponkan Razi Wildlife Sanctuary, experience mean annual temperatures of 6 °C and 3 °C, respectively. The temperatures on these two peaks drop during the coldest month to − 11 °C and − 14 °C.

The steep elevational gradient within 100 km from Putao northwards, the geological history, and the complex biogeography of the area^51^ translates into a large range of habitats and ecosystems, from moist evergreen lowland forest to alpine shrubs, glaciers and snowfields. This diversity of ecosystems increases the area’s conservation importance and makes it a globally unique biodiversity hotspot^51–53^.

### Land-cover mapping

We performed two single-date supervised land-cover classifications for the years 1989 and 2016 to map recent changes in 15 land-cover classes. These classifications were based on two adjacent Landsat 5 TM (1989) and two adjacent Landsat 8 OLI images (2016). The two Landsat images in each year had the same acquisition date and were captured during the dry season to minimize cloud cover interference (Table 1). All images were pre-processed by converting to Top of Atmosphere (TOA) reflectance and masking out clouds and cloud shadows using the FMASK algorithm^54^. We also used the C correction method to lessen the effects of topographic shadowing through topographic normalization^55^.

**Table 1:** Description of land-cover classes in the Hkakabo Razi Landscape, Myanmar.

Land-cover class	Description
Alpine vegetation	Vegetation on steep terrain above 3,000 m
Fir/Rhododendron	Fir forest and Rhododendron shrub above 2,500 m
Forest > 1,800 m	Forest with tree canopy cover > 80% between 1,800 and 2,500 m
Forest 600–1,800 m	Forest with tree canopy cover > 80% between 600 and 1,800 m
Forest < 600 m	Forest with tree canopy cover > 80% below 600 m or on flat or level terrain
Secondary forest 600–1,800 m	Degraded woody vegetation; logged areas; bamboo/rattan between 600 and 1,800 m
Secondary forest < 600 m	Degraded woody vegetation; logged areas; bamboo/rattan below 600 m or on flat or level terrain
Shrub/Bush	Regrowth early vegetation including fern and bushes
Grassland/Pasture	Grassland, pastures
Paddyfield	Rice paddy fields
Clearcut	Open areas without vegetation
Settlement	Human settlements, roads, buildings and man-made structures
Rock/Boulder	Rock, boulders including earth slides
Snow/Glacier	Snow, ice, glaciers
Streambed/Water	Bodies of fresh water including lakes, rivers; streambed and flooded areas

Training data for supervised land-cover classification were created by a combination of ground truth data and visual interpretation of fine-resolution imagery freely available through Google Earth. Ground truth data were collected by MSR, MK, and SCR. Visual interpretation of fine-resolution imagery was based on patch size and geometric shape, texture, colour, or vegetation phenology in cases where time series of reference imagery were available. We delineated separate sets of training and validation polygons for each of our 15 land-cover classes (Table 1). Training and validation data for forest classes in 2016 were largely identified based on ground truth data and prior knowledge of particular forest areas (field expeditions in December 2013/January 2014, January–March 2016). We created separate training data for forests in three elevation ranges (< 600 m, 600–1,800 m and > 1,800 m) due to expected differences in plant communities and associated spectral characteristics. Training and validation data for secondary (degraded) forest classes represented areas where early dry season canopy cover appeared to be less than 80%. Because intact forest canopies in the HRL are mostly closed^16^, we considered reduced canopy cover to be an indication of degradation. Importantly, the estimated extent of degraded forest, therefore, does not capture forest degradation that does not result in a reduction of canopy cover. Training and validation data for lowland forest were created only in areas less than 600 m in elevation that were visually identified as having flat or gently undulating terrain. The final training dataset consisted of approximately 70 polygons per land-cover class for a total of 1,080 polygons for 1989 and 1,047 for 2016. The separate validation dataset consisted of at least 20 additional polygons per land-cover class.

We also compiled several auxiliary data layers as predictor variables in our land-cover classification, because we expected different plant communities on north- vs. south-facing slopes, ridges vs. valleys, and steep slopes vs. flat flood plains. These included a 30-m digital elevation model (DEM) from NASA’s Shuttle Radar Topography Mission^56^, and several topographic measures derived from this DEM: slope, northness^57^, and topographic position index (TPI)^58^. As an auxiliary data source for distinguishing clearcuts from other bare ground (e.g., agriculture), we also calculated the change in Normalized Differenced Vegetation Index (NDVI)^59,60^ between 2013 and 2016. This data layer was created in Google Earth Engine^61^, a cloud-based analysis platform, by calculating the median NDVI value of all cloud-free Landsat pixels across each of two date ranges (31 January 2013 to 30 January 2014 and 31 January 2016 to 30 January 2017), and subtracting the 2016 median NDVI image from the 2013 image. This vegetation change data primarily spanned the > 3 years prior to our second classification date and was intended to identify cleared areas showing no signs of recent vegetation regrowth. Due to the limited number of Landsat images available from 1987 to 1990, we did not include NDVI change as a predictor variable for our 1989 classification.

We conducted a supervised classification using a Random Forest algorithm with the randomForest package^62,63^ in the R environment^64^. Random Forest is a non-parametric, machine learning classifier commonly used in remote sensing for classification of satellite or aerial images^65–67^. It is flexible concerning distributional assumptions about the training data, generally robust to over-fitting^68^, and thus, potentially better suited than non-parametric approaches for identifying target land-cover classes that include mixtures of spectral information^65,69^. To train the Random Forest classifier, we randomly selected 30 pixels from each training polygon for each land-cover class and used elevation, slope, northness, topographic position, single-band Landsat data (Landsat 8 OLI bands 2‒7; Landsat 5 TM bands 2‒6), and NDVI change (for 2016 only) as predictor variables. We set the following parameters for the Random Forest classifier: 500 decision trees, 2/3 of the training data sampled with replacement as the training set for each decision tree, and the number of predictor variables randomly sampled at each node split set to the square root of the number of variables. Due to the presence of cloud and cloud shadow gaps in our 2016 land-cover classification (up to 17% of Landsat Scene with clouds; Table S1), particularly at high elevations, we performed a secondary radar-based classification to fill gaps in our primary Landsat-based classification. Predictor variables for this secondary classification were Sentinel-1A VV and VH radar backscatter data and their ratio, collected in the Interferometric Wide (IW) swath acquisition mode. Sentinel-1A data were pre-processed by performing radiometric calibration, speckle noise filtering, Range-Doppler terrain correction, and conversion to decibel scaling using the SNAP 2.0 software^70^. Following gap-filling of the primary 2016 classification, we post-processed both the 1989 and 2016 classified images. We re-assigned forest pixels to the land-cover class corresponding with their actual elevation band if needed. Afterwards, we used a 3 × 3 majority filter to smooth isolated pixels that likely represent classification error.

We validated the accuracy of both single-date land-cover classifications (1989 and 2016) by randomly selecting 10 reference pixels from each of the 20 + validation polygons for each land-cover class in each year and comparing to the final classifications. Based on these reference points, we calculated standard accuracy metrics including an error matrix, producer’s accuracies (fraction of reference pixels for a given class that are correctly identified), user’s accuracies (fraction of pixels of a given class that are correct in the classified image), and overall accuracy following the recommendations of Olofsson et al.^71^.

### Landscape change analysis

As we were primarily interested in information on forest integrity to support conservation activities, we grouped land-cover classes into three main vegetation categories for subsequent analysis: (i) alpine vegetation; (ii) forest, which included forest at different elevation bands and fir/rhododendron; and (iii) shrubland, which comprised secondary forest and shrubs. Grassland/pasture, paddyfield, settlement and clearcut were also merged into “agriculture/developed”. We used these land-cover maps for constructing a transition matrix to summarize the area and percentage of land-cover change from 1989 to 2016 based on a pixel-by-pixel comparison^72^. To complement the transition matrix, we plotted the change values in a Sankey diagram (available at https://github.com/csaladenes/sankey, accessed 8 May 2019). Sankey diagrams, traditionally applied in the analysis of flows of energy and materials, have been recently employed for visualizing land-cover change transitions^73^.

In addition, we quantified forest fragmentation by computing the spatial density of forest cover, the Forest Area Density (FAD)^74,75^. FAD is defined as the proportion of the pixels in a surrounding fixed-area neighbourhood that is forest and measures both the area of continuous forest and of patches of forest separated by non-forest lands^76^. Each forest pixel is labelled as forest interior if the associated FAD ≥ 0.9^77^. To account for the scale-dependence of fragmentation, we evaluated FAD at five measurement scales defined by neighbourhood sizes equal to 4.41 ha (7 pixels × 7 pixels), 15.21 ha (13 × 13), 65.61 ha (27 × 27), 590.49 ha (81 × 81), and 5,314.41 ha (243 × 243) using GUIDOS Toolbox 2.8^78^. To quantify forest area gains and losses from 1989 to 2016, the maps of FAD in 1989 and 2016 were overlaid, on a pixel-by-pixel basis, upon the forest maps from 1989 and 2016.

### Spatial predictors of forest change

To understand factors that may explain the spatial dynamics of forest change, a multinomial logistic regression was performed^79,80^. We evaluated the effects of physical (slope, elevation, major landform, soil properties), environmental (temperature, precipitation, soil degradation) and geographical (distance from villages, from towns, from roads, from rivers) explanatory variables on the risk of forest transition between 1989 and 2016. Slope was derived from the digital elevation model (DEM) from NASA’s Shuttle Radar Topography Mission^56^. Soil properties were obtained from the World Reference Base (WRB), an international system for classification of soils^81^. Soil degradation and landform were based on a map produced by the UNEP Global Assessment of Human-Induced Soil Degradation (GLASOD). Soil degradation was identified based on expert opinion as all areas where human intervention had resulted in a decrease on the capacity of the soil to support life^82^. Mean temperature and annual precipitation for our analysis were based on the Global Climate Data WorldClim 2^83^. Euclidian distance from villages, towns and roads were calculated based on a national database from the MIMU-Myanmar Information Management Unit (https://themimu.info/ accessed 3 June 2019) and river data were accessed through the HydroSHEDS database (https://hydrosheds.cr.usgs.gov/hydro.php accessed 3 June 2019).

We used multinomial logistic regression models to predict transition probabilities between possible land-cover categories. In our case, there were three possible discrete outcomes for a pixel belonging to a given land-cover category (e.g., for forests: forest to forest; forest to shrubland; forest to agriculture/developed). We fit three separate multinomial logistic regression models, one for each land-cover category (forest, shrubland, agriculture/developed), and estimated the changes in transition probabilities due to slope, elevation, landform, soil properties, temperature, precipitation, soil degradation, and distances from villages, towns, roads, and rivers. In the first model, possible outcomes of the dependent variable were forest to forest, forest to shrubland and forest to agriculture/developed (n = 16,058,849). In the second model, possible outcomes were shrubland to shrubland, shrubland to forest and shrubland to agriculture/developed (n = 587,117). Lastly, in the third model possible outcomes were agriculture/developed to agriculture/developed, agriculture/developed to forest, and agriculture/developed to shrubland (n = 630,992). To deal with spatial autocorrelation, each of the models was run using a randomized subsample of 10% of the entire data set. This procedure was repeated 1,000 times and the mean of each coefficient estimate and 95% confidence intervals were calculated. The multinomial models were run in the nnet R package^84^. To aid the interpretation of the estimates of the multinomial logistic regression models (i.e., log of the odds ratio), we calculated the relative risk ratio (RRR) as exp(β)^85^. RRR > 1 indicates that the land-cover transition is more likely and RRR < 1 indicates that the land-cover transition is less likely (i.e., remaining in the current land cover is more probable).

## Results

### Land-cover mapping

**Figure 2:**
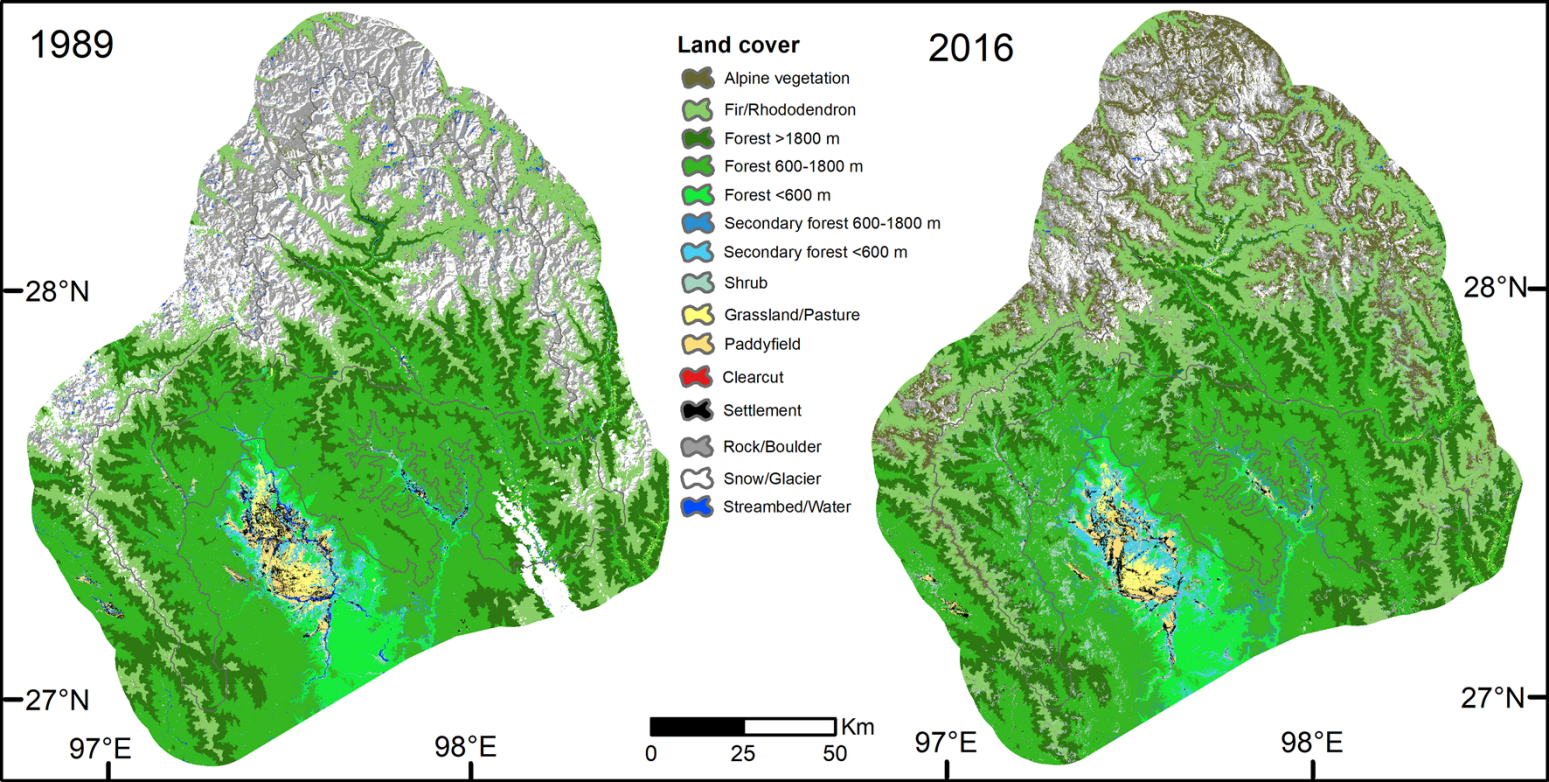
Land-cover classification for the Hkakabo Razi Landscape, northern Myanmar, for 1989 and 2016. The maps were produced in ArcGIS v. 10.6.1 (https://desktop.arcgis.com).

Two land-cover maps, each one for 1989 and 2016, were produced (Fig. 2) with overall accuracies of 90% and 85%, respectively (Table 2). The mean per-class user’s accuracy was 94% for 1989 and 91% for 2016. Forest at 600–1,800 m was occasionally misclassified in both years but more frequently in 2016. For the 2016 land-cover map, 30% of misclassified forest at 600–1,800 m was due to confusion with secondary forest, shrub/bush and clearcut. Shrub/bush was mistakenly classified in 10% of the cases as secondary forest at 600–1,800 m, and secondary forest at low elevation was confused in 14.5% of the cases with shrub/bush. Most classes were mapped with high accuracy in 1989, but for 2016 four classes were misidentified. Although clearcut was correctly identified in most cases, 34% of clearcut was misidentified. Similarly, secondary forest at low and mid-elevation and shrub/bush were misidentified in comparable magnitude (Table 2).

**Table 2:** User’s and producer’s accuracies of land-cover classes from the land-cover classification in 1989 and 2016. Overall accuracy was 0.90 for 1989 and 0.85 for 2016.

Land cover	1989		2016	
	User’s	Producer’s	User’s	Producer’s
Alpine vegetation	1.00	0.81	0.87	0.90
Fir/Rhododendron	0.97	0.99	0.91	1.00
Forest > 1,800 m	0.99	0.99	1.00	1.00
Forest 600–1,800 m	0.80	0.99	0.70	0.99
Forest < 600 m	0.88	0.91	0.86	0.96
Secondary forest 600–1,800 m	0.99	0.88	0.99	0.70
Secondary forest < 600 m	0.99	0.85	0.78	0.78
Shrub/Bush	0.97	0.87	0.71	0.77
Grassland/Pasture	0.96	0.90	1.00	0.96
Paddyfield	0.88	0.97	0.93	0.99
Clearcut	1.00	0.86	1.00	0.66
Settlement	0.97	0.93	0.98	0.96
Rock/Boulder	0.81	1.00	0.93	0.84
Snow/Glacier	1.00	0.94	0.93	0.97
Streambed/Water	0.88	1.00	1.00	1.00

Based on the error-adjusted estimates, forest covered most of the total area in 1989 (63.6%; Fig. 2, Table 3). Forest at 600–1,800 m was most common (24.5%), followed by forest > 1,800 m (17.5%) and fir/rhododendron (16.1%). Forest at low elevation (< 600 m) covered only 5.6% of the area. The extent of forest degradation, or areas showing canopy damage (i.e. secondary forest and shrub/bush), was low (5.4%). Grassland/pasture, paddyfield, clearcut and settlement (3.9%) occurred mainly at lower elevations in the Putao plains and around Naung Mung village. In 2016, forest also covered most of the area (57.8%). Secondary forest and shrub/bush covered 11.4%, mainly in the north and west of the Mali Raing region, west of the Putao plains, and east of Naung Mung. Clearcut areas covered 3.3%, whereas grassland/pasture, paddyfield and settlement covered almost the same area as in 1989.

**Table 3:** Error-adjusted estimated total area and 95% confidence intervals (CI) following Olofsson et al. (2014) and percentage of total area covered.

Land cover	1989			2016		
	Area (thousand ha)	± 95% CI (thousand ha)	Area covered (%)	Area (thousand ha)	± 95% CI (thousand ha)	Area covered (%)
Alpine vegetation	70.07	13.71	3.24	198.05	10.95	8.84
Fir/Rhododendron	347.36	9.19	16.06	386.29	15.81	17.24
Forest > 1,800 m	378.08	8.65	17.48	386.18	0.00	17.24
Forest 600–1,800 m	529.46	29.82	24.48	447.92	34.22	19.99
Forest < 600 m	121.07	19.60	5.60	73.76	5.86	3.29
Secondary forest 600–1,800 m	59.46	19.32	2.75	112.99	25.58	5.04
Secondary forest < 600 m	35.83	3.31	1.66	45.32	4.69	2.02
Shrub/Bush	22.34	7.58	1.03	96.02	18.78	4.29
Grassland/Pasture	26.28	3.63	1.22	18.91	1.33	0.84
Paddyfield	14.62	0.74	0.68	18.42	0.67	0.82
Clearcut	25.49	14.21	1.18	73.03	22.46	3.26
Settlement	17.88	1.06	0.83	16.63	1.49	0.74
Rock/Boulder	235.94	14.77	10.91	228.59	19.46	10.20
Snow/Glacier	269.33	10.29	12.45	124.22	5.48	5.54
Streambed/Water	9.92	0.47	0.46	14.07	0.00	0.63

### Landscape change from 1989 to 2016

**Figure 3:**
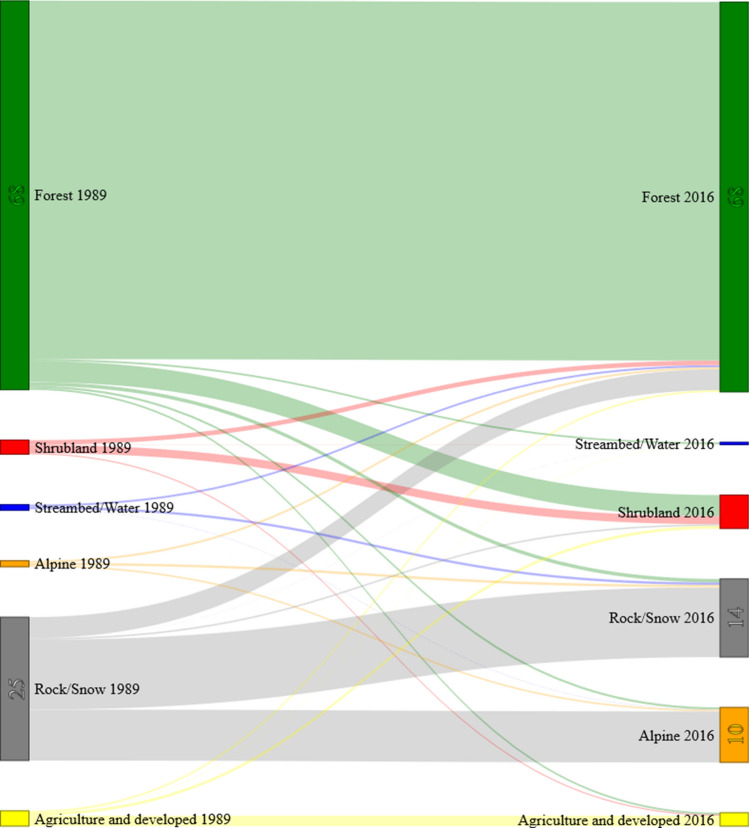
Sankey diagram showing the relative changes from 1989 to 2016 for each land-cover class.

**Figure 4:**
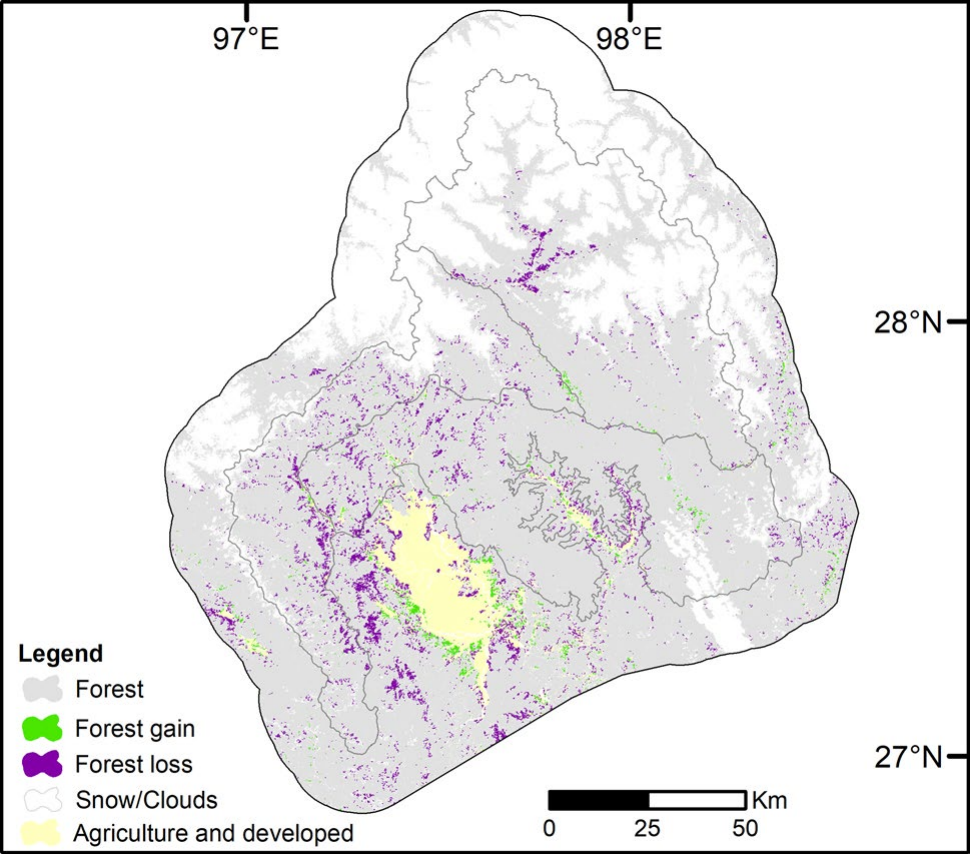
Map indicating spatially explicit forest loss and gain in the Hkakabo Razi Landscape from 1989 to 2016. The gray lines represent the proposed and designated protected areas. The map was produced in ArcGIS v. 10.6.1 (https://desktop.arcgis.com).

The analysis of 27 years of land-cover change indicated that the deforestation rate for the study period was 2.7%. Forest cover decreased from 1,375 thousand ha in 1989 to 1,294 thousand ha in 2016 (annual average rate = 0.225%). The loss of forest was mainly due to an increase in shrub/bush and secondary forest (5.9%) from 84 thousand ha in 1989 to 127 thousand ha in 2016 (Fig. 3). Agriculture/developed areas (i.e. grassland/pasture, paddyfield, clearcut and settlement) increased 1.7%, mainly driven by an increase of clearcut areas (Table 3, Table S2). Forest gain was mainly concentrated near agriculture/developed areas, whereas forest loss tended to follow the distribution of shrub/bush and secondary forest in 2016 occurring mainly in the southern extension, the proposed protected area (Fig. 4).

**Figure 5:**
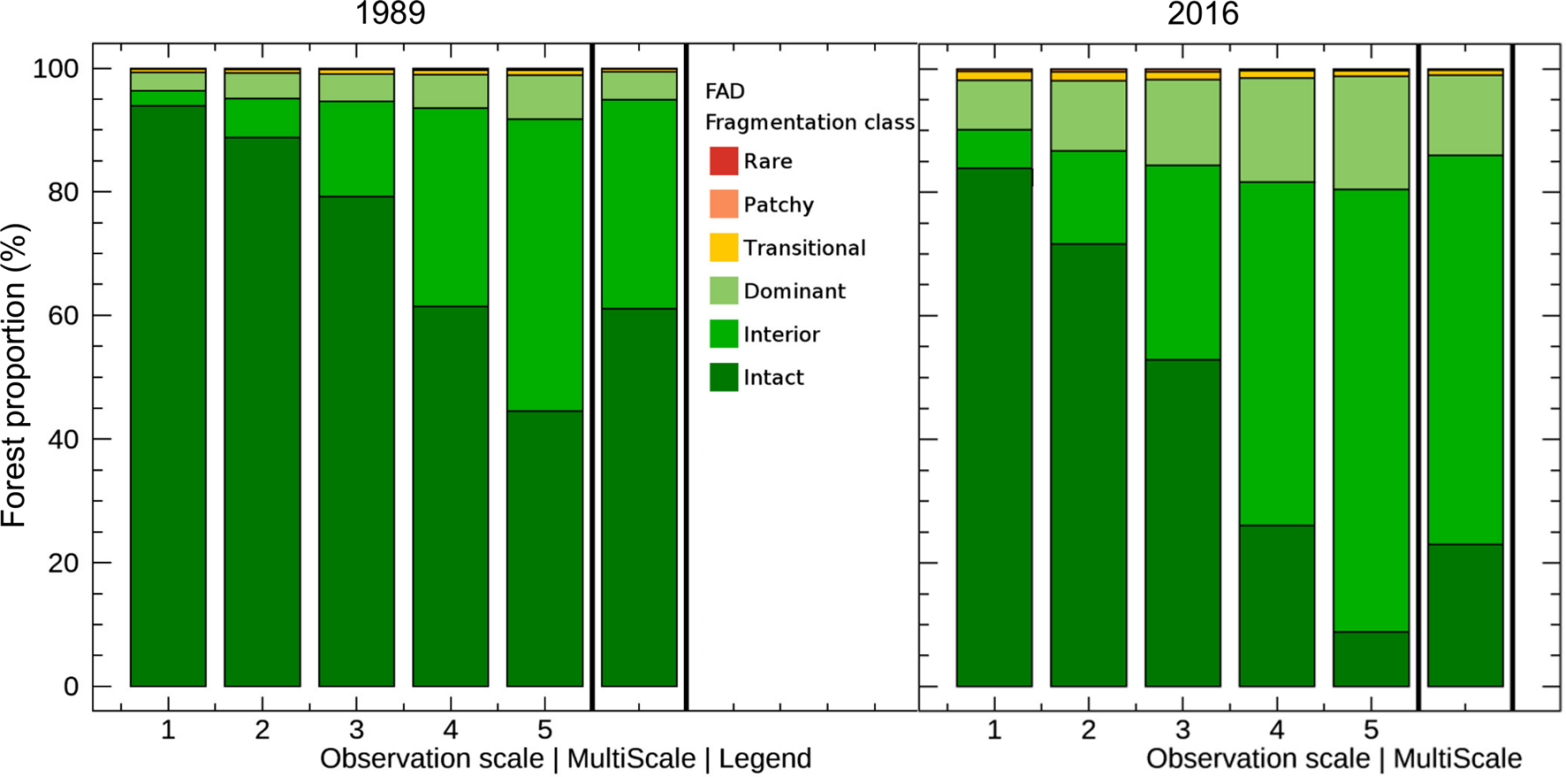
Proportion of forest for each fragmentation class at five different observational scales for 1989 and 2016.

The net loss of forest interior area was at least 107 thousand ha, regardless of the scale of analysis, with a maximum loss of 155 thousand ha for the 590.5-ha neighbourhood size (Table 4). The rate of loss of forest interior area increased monotonically with neighbourhood size (except for the largest one) and was approximately 2 to 3 times larger than the rate of loss of total forest area. Even though deforestation has occurred in the HRL, forest interior was prevalent with little fragmentation (Fig. 5).

**Table 4:** Change in forest interior area from 1989 to 2016 at different neighbourhood sizes in the Hkakabo Razi Landscape, Myanmar.

Neighborhood size (ha)	Forest interior			
	1989	2016	Change	
	(thousand ha)	(thousand ha)	(thousand ha)	(%)
4.41	903.5	796.9	− 106.6	− 11.8
15.2	892.0	766.0	− 126.0	− 14.1
65.6	887.4	745.7	− 141.6	− 16.0
590.5	877.2	721.8	− 155.4	− 17.7
5,310.4	860.7	711.4	− 149.4	− 17.4

### Spatial predictors of forest change

**Figure 6:**
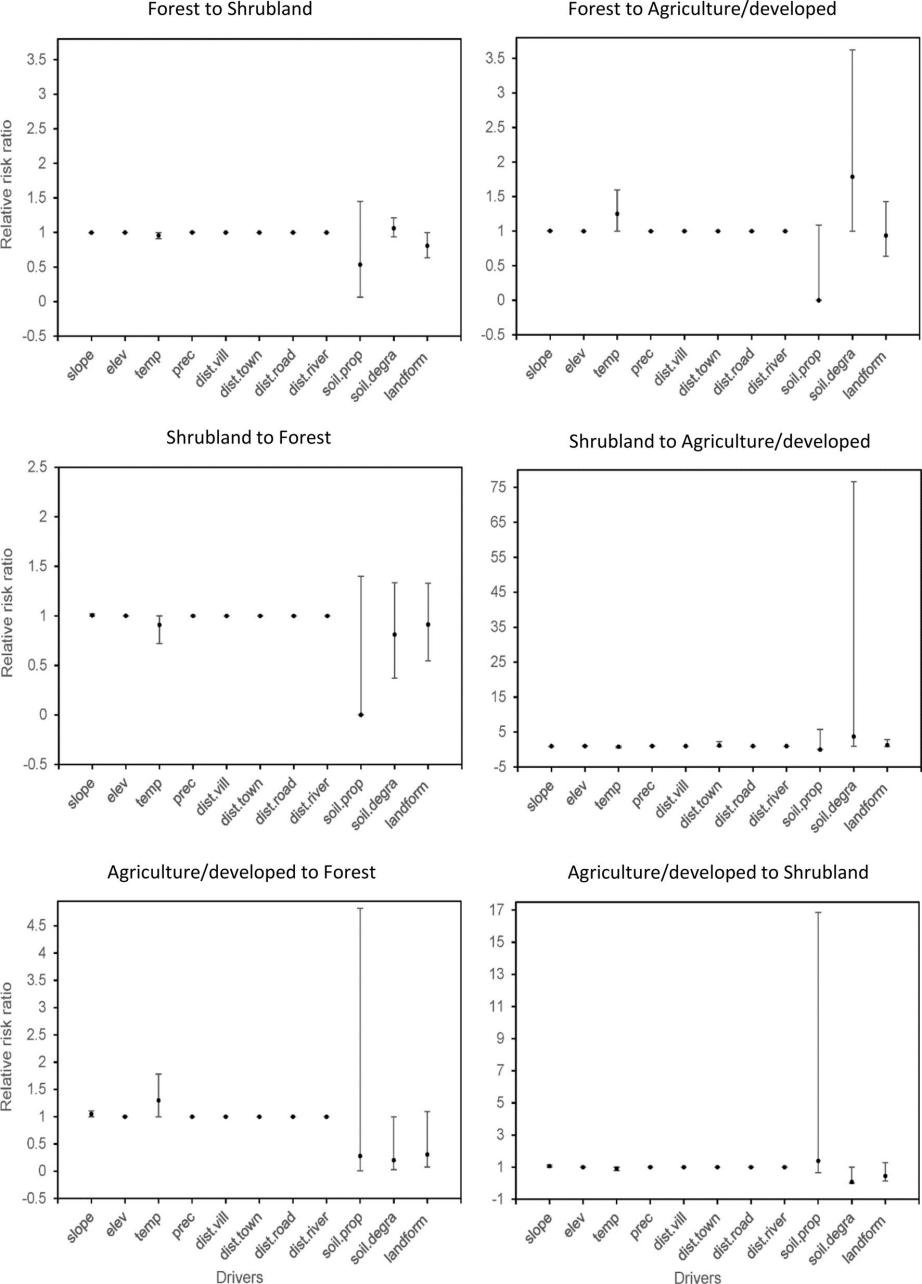
Relative risk ratios (RRR) of the multinomial logistic regression models predicting land-cover transitions between 1989 and 2016 from physical, environmental and geographical variables.

The multinomial logistic regression models showed that physical and environmental variables were the main drivers of either remaining in the current land-cover class or transitioning to another class (Fig. 6, Table S3). Forest was most likely to remain as forest (94% probability) and its persistence was related to slope and landform (i.e. steep land). Forest conversion, although less likely (< 6%), was predicted by soil degradation and temperature. Areas with mild temperatures had a higher risk of forest conversion and becoming agriculture/developed land. Shrubland was most likely to remain shrubland (59%) and its persistence was associated with soil conditions, landform and temperature. Shrubland conversion to agriculture/developed land, although less likely (8%), was predicted by soil degradation and distance from towns. Shrubland close to towns, in valleys or plain areas, and with degraded soils were more likely to become agriculture/developed land. Agriculture/developed land was most likely to remain stable (68%) and its persistence was associated with soil conditions. Conversion from agriculture/developed land to forest (12%) was predicted by temperature. Low temperature areas were more likely to become forest.

## Discussion

Over 80% of the HRL is covered by natural areas, from which forest is the most prevalent land cover. Agriculture/developed land was low (6%) and occurred mainly at lower elevations in the larger valleys around Putao and Naung Mung. Although the extent of forest degradation represented by shrubs and secondary forest increased from 1989 to 2016, the deforestation rate over the last three decades was low (annual average rate: 0.225%). The annual deforestation rate increased slightly compared with the deforestation rate reported for the region between 1991 and 1999 (annual average rate: 0.01%)^39^. However, the apparent increase in deforestation should be considered with caution. The period, the extent of the study area, the baseline dataset and the analytical methods diverged among studies making a plain comparison difficult. Nonetheless, the increased deforestation rate might indicate that forest loss surged since 2000.

Forest loss was mainly located around the Naung Mung plains, along major roads, west of the Putao plains, and the western area of the proposed southern extension. This forest loss was due to the conversion from forest to shrub/bush and secondary forest indicating clearcuts, logging, and extraction of resources. Interestingly, we found a large part of forest loss areas replaced by shrub/bush and secondary forest on high altitude areas. This might be explained by a large wildfire that occurred in 1998 in the Hkakabo Razi National Park between Tazungdam and Tahaundam (own unpublished data Thein Aung). During the wildfire, large tracts of forest burned down. In 2001, fern covered the burned sites, in 2014 shrub/bush vegetation of roughly 5 m height emerged. Only minor areas close to small settlements have been used continuously since 2001, while a majority of the fern-covered areas were replaced by early succession vegetation, indicating regrowth as vegetation is gradually recovering from strong past disturbance (i.e., wildfires).

Compared to other forested regions in Myanmar, the annual deforestation rate of the HRL is still very low. For example, the deforestation rate at Chattin Wildlife Sanctuary from 1973 to 2005 was 1.86%^86^, an order of magnitude higher than for the HRL. Although, the annual deforestation rate for all forests in the country was between 0.55% between 2002 and 2014^16^ and 0.62% from 2005 to 2016^87^, some areas have experienced annual deforestation rates above the country’s average. Only 38% of the country’s forests can be considered intact with canopy cover > 80%^16^.

Our results show that even though some deforestation occurred in the HRL, forest interior was prevalent with little fragmentation. This suggests that the remoteness of the area and the difficult access have played a role in safeguarding these large tracts of forest. So far, the only access is by air through Putao airport or the Myitkyina-Putao road. However, the recent paving of the Myitkyina-Putao road has reduced the time of transportation to less than two days instead of up to seven days of travel prior to 2014. The latent plans to connect the existing dirt road Putao-Pangnamdim with Yunnan/China would allow easy access to the site and pave the way for subsequent forest losses. Compared to the access from within Myanmar, accessibility from abroad is relatively easy. The lack of efficient in-country accessibility into the HRL and particularly the Hkakabo Razi National Park is regarded as unjust by the local communities (mainly Rawan, Lisu, and Tibetans) and causes major irritations (personal communication, September 2019 with the Rawan Cultural and Literary Organisation Putao). Local Rawan organisations in the HRL learned during the opening up of Myanmar since 2015 that accessibility would allow extraction of natural resources; major irritations come from who has the right to use and extract natural resources in HRL. The lack of efficient in-country accessibility has already resulted into tensions between conservation stakeholders, local organisations representing the Rawan, other local ethnic/religious groups entering into HRL, and government organizations. Addressing and balancing accessibility and extraction of natural resources are central for future management plans and conservation measures^42^ to ensure the protection of these large tracts of forest interior and thus the long-term future of the country's forests.

Regression analyses showed that remaining in the current land-cover class was more likely than land conversion. Physical variables (i.e., slope and major landform) were associated with stable forest. As has been shown previously, forests that are difficult to reach have low probability of conversion, as they are located at the top edge of mountains or along steep cliffs^80,88–90^. These results highlight that although major land conversion is occurring in other areas of the country^16^, the HRL has experienced low human impact and may be especially vulnerable to forest changes if sustainable forest policies and management are not put in place. It is important to note, though, that addressing the risk of deforestation does not only encompasses physical, environmental and geographical variables, but also interrelationships among political, institutional, economic, and ethnic and cultural factors should be considered^21,91^.

Based on our assessments, the forests of the HRL have experienced low human impact and still constitute large tract of contiguous forest interior. Therefore, the HRL should be regarded as of high integrity as for 2016 sensu Belle et al.^45^ and should have high priority for protection. However, factors threatening the forest are emerging slowly, but steadily and with potential for an exponential increase. Thus, the HRL needs an inclusive forests management plan and effective conservation strategies to support biodiversity, guarantee the sustainable subsistence of local communities, and prevent irreversible forest loss and degradation.

## Supplementary information

Supplementary Information

## Data Availability

The datasets generated during the current study are available from the corresponding author upon request.
