# Supplementary material for: Hkakabo Razi landscape as one of the last exemplar of large contiguous forests
Source: Sci Rep. 2020 Aug 19;10:14005. doi: 10.1038/s41598-020-70917-y (PMC7438525; doi:10.1038/s41598-020-70917-y)
Supplement: Supplementary file 1 — Supplementary Information [file 41598_2020_70917_MOESM1_ESM.docx]

Hkakabo Razi Landscape as one of the last exemplar of large contiguous forests

**Marcela Suarez-Rubio**^1*^, **Grant Connette**^2^, **Thein Aung**^3^, **Myint Kyaw**^4^, **Swen C. Renner**^5^

^1^Institute of Zoology, University of Natural Resources and Life Sciences, Gregor-Mendel-Strasse 33, A-1180 Vienna, Austria; ORCID: 0000-0002-0596-2626

^2^Conservation Ecology Centre, Smithsonian Conservation Biology Institute, 1500 Remount Road, Front Royal, VA 22630, USA; ORCID: 0000-0002-9424-6591

^3^Myanmar Bird and Nature Society, 221/223 Shwegondine Road, Yangon, Myanmar

^4^Mount Popa National Park Headquarters, Popa, Myanmar; ORCID: 0000-0003-3881-2402

^5^Ornithology, Natural History Museum, Burgring 7, A-1010 Vienna, Austria; ORCID: 0000-0002-6893-4219

^*^Corresponding author: [marcela.suarezrubio@boku.ac.at](mailto:marcela.suarezrubio@boku.ac.at); Tel: +43 1 47654 83316

# Supplementary Material

Table S1. Landsat scenes and tiles used in the analysis

| Path/Row | Date | Cloud Cover (%) | Scene ID |
| --- | --- | --- | --- |
| 133/041 | 25 February 1989 | 1.67 | LT51330411989056BKT01 |
| 133/040 | 25 February 1989 | 17.12 | LT51330401989056BKT01 |
| 133/041 | 2 November 2016 | 3.27 | LC81330412016307LGN00 |
| 133/040 | 2 November 2016 | 1.56 | LC81330402016307LGN00 |

Table S2. Land-cover change matrix from 1989 (rows) to 2016 (columns) using a standard post-classification approach. Data show land area (ha) and percentage of total area (%) of grouped land-cover classes in the Hkakabo Razi Landscape, Myanmar

| 2016  1989 | Alpine | Forest | Shrub | Agriculture/  developed | Rock/Snow | Streambed/  Water |
| --- | --- | --- | --- | --- | --- | --- |
| Land area (ha) |  |  |  |  |  |  |
| Alpine | 6394 | 7629 | 49 | 0 | 8991 | 65 |
| Forest | 8367 | 1358404 | 79755 | 7078 | 13598 | 7125 |
| Shrub | 0 | 17585 | 30846 | 4280 | 0 | 582 |
| Agriculture/developed | 0 | 5507 | 10652 | 39118 | 1 | 945 |
| Rock/Snow | 193180 | 79325 | 5836 | 17 | 264528 | 370 |
| Streambed/Water | 576 | 6738 | 797 | 2004 | 4395 | 7298 |
| Percentage (%) |  |  |  |  |  |  |
| Alpine | 0.29 | 0.35 | 0.00 | 0.00 | 0.41 | 0.00 |
| Forest | 0.39 | 62.54 | 3.67 | 0.33 | 0.63 | 0.33 |
| Shrub | 0.00 | 0.81 | 1.42 | 0.20 | 0.00 | 0.03 |
| Agriculture/developed | 0.00 | 0.25 | 0.49 | 1.80 | 0.00 | 0.04 |
| Rock/Snow | 8.89 | 3.65 | 0.27 | 0.00 | 12.18 | 0.02 |
| Streambed/Water | 0.03 | 0.31 | 0.04 | 0.09 | 0.20 | 0.34 |

Table S3. Parameter estimates of the multinomial logistic regression models predicting land-cover transitions between 1989 and 2016

| Variables | Coefficient | Std. Error | 95% CI | |
| --- | --- | --- | --- | --- |
| **Forest to Shrubland** | |  |  |  |
| intercept | -2.832 | -2.828 | -5.813 | 0.000 |
| slope | -0.001 | -0.001 | -0.003 | 0.000 |
| elev | 0.000 | 0.000 | 0.000 | 0.001 |
| temp | -0.045 | -0.045 | -0.096 | 0.000 |
| prec | 0.001 | 0.001 | 0.000 | 0.002 |
| dist.vill | 0.000 | 0.000 | 0.000 | 0.000 |
| dist.town | 0.000 | 0.000 | 0.000 | 0.000 |
| dist.road | 0.000 | 0.000 | 0.000 | 0.000 |
| dist.river | 0.000 | 0.000 | 0.000 | 0.000 |
| soil.prop | -0.626 | -0.631 | -2.747 | 0.371 |
| soil.degra | 0.059 | 0.058 | -0.064 | 0.191 |
| landform | -0.213 | -0.214 | -0.452 | 0.000 |
| **Forest to Agriculture/developed** | |  |  |  |
| intercept | -4.067 | -4.076 | -8.617 | 0.000 |
| slope | 0.004 | 0.004 | 0.000 | 0.009 |
| elev | 0.000 | 0.000 | -0.001 | 0.000 |
| temp | 0.224 | 0.225 | 0.000 | 0.467 |
| prec | -0.001 | -0.001 | -0.002 | 0.000 |
| dist.vill | 0.000 | 0.000 | 0.000 | 0.000 |
| dist.town | 0.000 | 0.000 | 0.000 | 0.000 |
| dist.road | 0.000 | 0.000 | 0.000 | 0.000 |
| dist.river | 0.000 | 0.000 | -0.001 | 0.000 |
| soil.prop | -36.598 | -36.763 | -81.312 | 0.084 |
| soil.degra | 0.581 | 0.584 | 0.000 | 1.287 |
| landform | -0.065 | -0.065 | -0.454 | 0.357 |
| **Shrubland to Forest** |  |  |  |  |
| intercept | 1.045 | 1.037 | 0.000 | 3.996 |
| slope | 0.006 | 0.006 | 0.000 | 0.018 |
| elev | 0.001 | 0.001 | 0.000 | 0.003 |
| temp | -0.095 | -0.095 | -0.329 | 0.000 |
| prec | 0.000 | 0.000 | 0.000 | 0.001 |
| dist.vill | 0.000 | 0.000 | 0.000 | 0.000 |
| dist.town | 0.000 | 0.000 | 0.000 | 0.000 |
| dist.road | 0.000 | 0.000 | 0.000 | 0.000 |
| dist.river | 0.000 | 0.000 | 0.000 | 0.000 |
| soil.prop | -7.629 | -6.650 | -39.721 | 0.338 |
| soil.degra | -0.210 | -0.212 | -0.994 | 0.289 |
| landform | -0.091 | -0.093 | -0.606 | 0.284 |
| **Shrubland to Agriculture/developed** | | |  |  |
| intercept | 3.338 | 3.255 | 0.000 | 11.561 |
| slope | -0.031 | -0.031 | -0.097 | 0.000 |
| elev | 0.000 | 0.000 | 0.000 | 0.001 |
| temp | -0.193 | -0.190 | -0.666 | 0.000 |
| prec | 0.001 | 0.001 | -0.001 | 0.000 |
| dist.vill | 0.000 | 0.000 | 0.000 | 0.000 |
| dist.town | 0.151 | 0.133 | 0.000 | 0.812 |
| dist.road | 0.000 | 0.000 | 0.000 | 0.000 |
| dist.river | 0.000 | 0.000 | 0.000 | 0.000 |
| soil.prop | -7.705 | -4.599 | -29.300 | 1.753 |
| soil.degra | 1.318 | 1.304 | 0.000 | 4.340 |
| landform | 0.261 | 0.258 | -0.109 | 1.037 |
| **Agriculture/developed to Forest** | |  |  |  |
| intercept | -2.579 | -2.544 | -6.465 | 0.000 |
| slope | 0.049 | 0.049 | 0.002 | 0.101 |
| elev | 0.001 | 0.001 | 0.000 | 0.002 |
| temp | 0.262 | 0.260 | 0.000 | 0.577 |
| prec | -0.001 | -0.001 | -0.001 | 0.000 |
| dist.vill | 0.000 | 0.000 | 0.000 | 0.000 |
| dist.town | 0.000 | 0.000 | 0.000 | 0.000 |
| dist.road | 0.000 | 0.000 | 0.000 | 0.000 |
| dist.river | 0.000 | 0.000 | 0.000 | 0.000 |
| soil.prop | -1.275 | -1.283 | -4.738 | 1.573 |
| soil.degra | -1.597 | -1.599 | -3.505 | 5.846E-06 |
| landform | -1.180 | -1.177 | -2.564 | 0.089 |
| **Agriculture/developed to Shrubland** | | |  |  |
| intercept | 1.270 | 1.295 | 0.000 | 4.046 |
| slope | 0.061 | 0.061 | 0.001 | 0.124 |
| elev | -0.001 | -0.001 | -0.003 | 0.000 |
| temp | -0.086 | -0.089 | -0.238 | 0.000 |
| prec | 0.001 | 0.001 | 0.000 | 0.002 |
| dist.vill | 0.000 | 0.000 | 0.000 | 0.000 |
| dist.town | 0.000 | 0.000 | 0.000 | 0.000 |
| dist.road | 0.000 | 0.000 | 0.000 | 0.000 |
| dist.river | 0.000 | 0.000 | 0.000 | 0.000 |
| soil.prop | 0.327 | 0.280 | -0.438 | 2.824 |
| soil.degra | -2.592 | -2.594 | -5.533 | 0.000 |
| landform | -0.797 | -0.794 | -1.972 | 0.247 |
